# Prehabilitation to improve function after autologous stem cell transplantation: A pilot randomized controlled trial (PIRATE)

**DOI:** 10.1007/s00520-025-09179-1

**Published:** 2025-02-07

**Authors:** Amy M. Dennett, Judi Porter, Stephen B. Ting, Nicholas F. Taylor

**Affiliations:** 1 School of Allied Health, Human Services and Sport, La Trobe University, Bundoora, Australia; 2 Allied Health Clinical Research Office, Eastern Health, Box Hill, Australia; 3 Institute for Physical Activity and Nutrition (IPAN), School of Exercise and Nutrition Sciences, Deakin University, Geelong, Australia; 4 Department of Clinical Haematology, Eastern Health, Box Hill, Australia; 5 Eastern Health Clinical School, Monash University, Box Hill, Australia

**Keywords:** Prehabilitation, Exercise, Nutrition, Supportive care, Cancer, Bone marrow transplant

## Abstract

**Purpose:**

Exercise and nutrition interventions are not part of routine care for those undergoing autologous stem cell transplant (autoSCT). We aimed to explore estimates of effect, safety and feasibility of multidisciplinary prehabilitation for improving physical capacity after autoSCT.

**Methods:**

This single-blinded, parallel, two-armed pilot randomized trial included adults receiving autoSCT. Participants were randomized to twice-weekly, supervised, tailored exercise and fortnightly telephone-based nutrition education, for up to 8-weeks prior to autoSCT (*n* = 11) or usual care (*n* = 11). Blinded assessments occurred at baseline (T0), pre-transplant (T1), and 4-weeks post-transplant (T2). The primary outcome was physical capacity (6-min walk test). Secondary measures included recruitment rate, adverse events, exercise adherence, physical status, nutritional status, health-related quality of life, and health service outcomes.

**Results:**

Positive estimates of effect for walking capacity in favour of the experimental group were demonstrated at T2 (MD + 141 m, 95% CI 24 to 257 m). There was high recruitment (81%) and adherence and no major adverse events. At T2 there were large estimates of effect favoring the experimental group for higher bodyweight, and less dyspnea and gastrointenstinal symptoms. There were no between-group differences in other outcomes.

**Conclusion:**

Prehabilitation is safe, feasible and may improve walking capacity after autoSCT. Findings support a future fully-scaled trial of prehabilitation for autoSCT.

**Trial registration:**

Australian New Zealand Clinical Trials Registry ACTRN12620000496910. Registered April 20, 2020.

**Supplementary Information:**

The online version contains supplementary material available at 10.1007/s00520-025-09179-1.

## Background

Autologous stem cell transplantation (autoSCT) improves survival and provides long term disease control for haematological malignancies [1]. Despite advances in survivorship, people undergoing autoSCT experience adverse treatment effects including fatigue, deconditioning, and psychological distress leading to ongoing functional decline and poor quality of life [2–4]. AutoSCT recipients are also at high risk of developing secondary cancers, and experience high rates of hospital readmission [1, 5].

Exercise and nutrition interventions are strongly recommended during cancer treatment, including autoSCT [6]. These interventions are safe and reduce fatigue, weight-loss and improve function and quality of life in people receiving autoSCT [7–13]. However, the optimal time to provide intervention is unknown, with most trials targeting the peri and post-transplant period [7–9]. Exercise and nutrition interventions delivered pretransplant (prehabilitation) may elicit superior benefits by building functional reserves prior to transplant. Exercise prior to stem cell transplant may result in better outcomes than post-transplant including reduced hospital length of stay of 2 days [8, 9]. No trials currently have evaluated combined supervised exercise and nutrition prehabilitation in autoSCT which may provide additional benefit to prevent post-transplant malnutrition [14]. Therefore, a pilot evaluation of combined exercise and nutrition prehabilitation in autoSCT is needed to determine if a full-scale randomized controlled trial is warranted [15].

The primary aim of this pragmatic pilot trial was to explore efficacy and provide estimates of effect of multidisciplinary prehabilitation on post-autoSCT physical capacity. Secondary aims were to explore feasibility evaluated through safety and exercise adherence; and efficacy on physical and nutritional status, health-related quality of life, and health service outcomes.

## Methods

### Study design

A prospective, pragmatic, parallel, single-blind, pilot randomized controlled trial was conducted at a large public tertiary hospital in metropolitan Melbourne, Australia from April 2021 to March 2023. Due to frequent changes in COVID-19 restrictions during this time, recruitment was paused for four-months. Final participants were recruited in August 2022. However medical delays to completing the transplant procedure for the final four participants resulted in extended duration of the trial. The protocol for this study has been published [16] and was registered prospectively (ACTRN12620000496910). Procedures were approved by the hospital’s ethics committee (E20/003/61055) and are reported consistent with the CONSORT statement for pilot and feasibility trials [17].

Participants were randomly allocated to either an experimental or usual care control group after baseline assessment according to an online computer-generated randomization program, using permuted blocks without stratification. Allocations were prepared prior to trial commencement by an independent researcher with no role in subject recruitment or administration of interventions. The trial coordinator allocated participants after baseline assessment by contacting the independent researcher by email for allocation. Outcome measures were performed at baseline (T0, week 0), the end of the intervention, *before* autoSCT (T1, approximately week 8) and 1-month *after* autoSCT (T2, week 12) by an assessor blinded to group allocation.

### Participants

Participants: were aged 18 years and over; had a hematological malignancy and were waitlisted for autoSCT. Participants were excluded if they: were medically unfit to exercise as determined by a clinician based on recommendations (e.g. unstable bony metastases, haemodynamic instability) [18]; had low physical performance status (Eastern Cooperative Oncology Group (ECOG) score > 2) [19]; or cognitive impairment precluding ability to provide written, informed consent. Patients were screened consecutively and all patients meeting inclusion were approached for enrolment by the nurse coordinator. T0 measurements were obtained following the consent process.

### Intervention

All participants received usual care. Usual care in this trial comprised an assessment with a physiotherapist (60 min), who provided standardized written instructions with guidelines for exercise [20–22] and a referral to a cancer rehabilitation program *after* autoSCT. Both groups received a comprehensive dietary assessment using the Australian Eating Survey [23] facilitated by the blinded assessor at T0 and T2. Collection of dietary data from control group participants was in addition to methods reported in the published protocol and enabled comparison of between-group intake [24]. Participants received their usual medical care, which may have included adjuvant chemotherapy, radiotherapy, inpatient admission, outpatient appointments and general practitioner visits.

### Experimental group (prehabilitation)

In addition to usual care, participants allocated to the experimental group received an individualized exercise program. Participants performed twice-weekly, 60-min exercise sessions individually at home, supervised by a cancer physiotherapist for up to 8-weeks pre-transplant (Supplementary File 1). To aid adherence, session times were negotiated individually with participants. Exercise sessions comprised moderate-intensity aerobic and resistance exercise [6, 16]. Participants completed approximately 25 min each aerobic and resistance exercise, 5-min flexibility/balance training and 5-min warm up and cool down. Initially, participants aimed to exercise at a BORG RPE of 3 (moderate), progressing to a 5–6 (hard) by week 8. For resistance exercise, weights were progressed once a participant achieved 2 to 3 sets of 10–12 repetitions. Resistance exercise included body-weight upper and lower body exercises, free weights, and resistance bands. Aerobic exercise included walking or portable stepper machine. Participants were encouraged to complete an additional once-weekly unsupervised 30-min aerobic exercise session independently and provided with a Fitbit Inspire device to assist compliance with exercise guidelines [6].

At baseline, all participants were categorized using GLIM Criteria to identify malnutrition risk [25]. Participants allocated to the experimental group received a further 60-min comprehensive consultation with an Accredited Practising Dietitian who provided written information and tailored medical nutrition therapy via fortnightly phone or video calls pre-transplant (up to 4 review sessions). These sessions focused on supporting oral intake and maintaining nutritional status pre-autoSCT and managing gastrointestinal symptoms post-transplantation [26].

### Outcomes

Patient outcomes were measured at T0, T1 and T2. Hospital length of stay was recorded at T2, emergency department, Symptom and Urgent Review Clinic (SURC) presentations and hospital readmissions were recorded at week 25 from hospital databases.

### Primary outcome: physical capacity at T2

Physical capacity was measured as walk distance (m) using the 6-min walk test. The 6-min walk test is a valid and reliable measure of physical function in cancer survivors [27].

### Secondary outcomes

Adverse events were categorised as minor or serious and graded according to Common Terminology Criteria for Adverse Events (CTCAE V5) [28]. Reasons for non-adherence to exercise or non-completion of the program, including both medical (e.g. fatigue, unwell) and psychosocial reasons and complications related to transplant procedure were recorded.

Physical status measures included: moderate-intensity physical activity and sedentary time measured by a tri-axial accelerometer-based activity monitor (ActivPAL™, PAL Technologies Ltd., Glasgow, UK) [29], self-reported physical activity self-efficacy [30], C-reactive protein, time to stem cell engraftment (neutrophils > 0.5 × 10^9^/L for three days without support and platelets > 50 × 10^9^/L for five days without transfusion) [31] and handgrip strength [32]. Nutritional status was assessed by Patient-Generated Subjective Global Assessment (PG-SGA) [33]. The European Organization for Research and Treatment of Cancer QoL Questionnaire-C30 (EORTC-QLQ C30) [34] and high dose chemotherapy supplement (EORTC-HDC 29) assessed quality of life [35].

### Data analysis

Anticipating a clinically significant change of 41 m on the 6-min walk test [27], a minimum total sample of *n* = 12 to produce a one-sided 80% confidence limit that would exclude an effect of 0.5 would be required. A one-sided 90% confidence interval would require a larger sample size of *n* = 28. Therefore, we recruited 22 participants for this pilot study [36]. An estimate of effect meeting or exceeding clinical significance would justify proceeding to a larger trial.

The primary outcome was analysed using linear mixed effects models. This method allowed participants to have missing observations at certain time points. Multiple imputation was not used as the assumption of data missing at random was not met [37]. Linear mixed effects models were also applied for analysis of continuous secondary outcomes. Physical activity data were included where there were at least 3 full (24 h) days of data [38]. Number of adverse events and intervention adherence was described. Proportion of participants meeting physical activity [39] and nutritional intake [24] guidelines was described and compared between groups with a risk ratio. The number of emergency department, SURC presentations and hospital admissions were described due to the low number of expected events. Differences in hospital length of stay and time to engraftment were calculated using Mann–Whitney U tests. All available data were analyzed as per group allocation using intention-to-treat principles, regardless of adherence [40]. Data were analyzed using IBM SPSS v29.

### Impact of COVID-19 pandemic

This trial commenced April 2021 and was completed in March 2023. Modifications were made in response to frequent changes in COVID-19 restrictions during the trial period, as Melbourne was the most locked-down city in the world (263 days) [41]. Fewer autoSCT were completed and there were periods where non-essential care, including research, was not permitted. The intervention was converted to a home setting [16], with most exercise sessions conducted in-person, with some completed via videocall. All nutrition interventions were conducted by telehealth (phone or video call). Some physical outcome measurements (6MWT and handgrip strength) were unable to be completed and nutrition outcomes relied on self-report (bodyweight) or video call (PG-SGA).

## Results

**Fig. 1:**
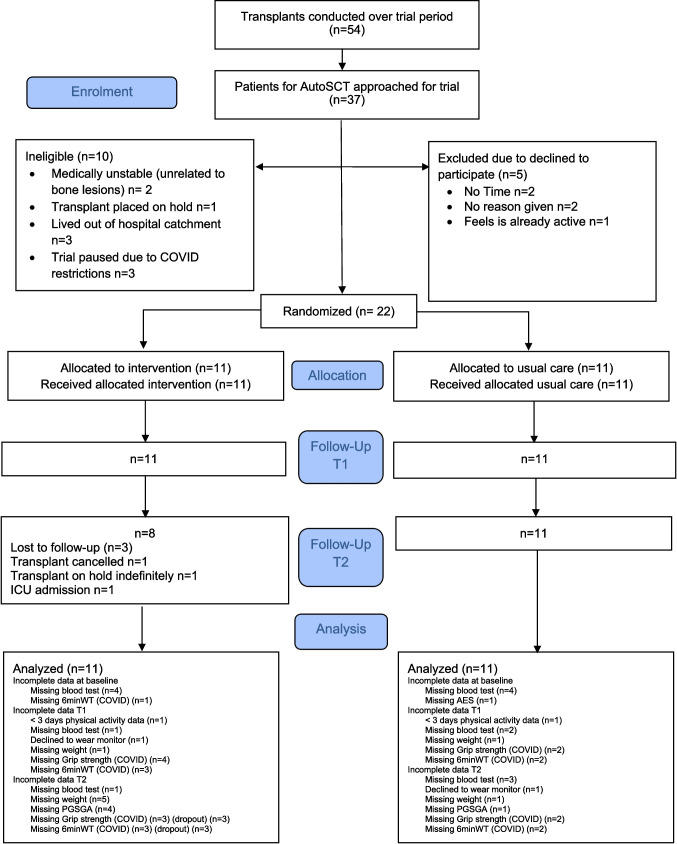
Flow of participants through trial. Abbreviations: 6mWT 6 min walk test; PG-SGA Patient Generated Subjective Global Assessment; AES Australian Eating Survey; ICU Intensive Care Unit

Thirty-seven patients were approached (Fig. 1). Of those, 10 were ineligible. Twenty-two participants consented (recruitment rate 81% of eligible patients) and were allocated. All participants received the intervention as allocated. One participant had their transplant cancelled and another put on hold indefinitely at the end of the intervention period. On average, participants were aged 63 years (range 36 to 71), male (*n* = 15, 68%) diagnosed with multiple myeloma (*n* = 20, 91%) and were recruited, on average, 13 weeks prior to autoSCT (Table 1). Half the participants had stable bony lesions (*n* = 11). One usual care group participant was severely malnourished at baseline. Participants were generally well matched at baseline. However, participants in the experimental group were younger, and more time had elapsed since diagnosis.

**Table 1:** Baseline characteristics

		Randomized	
	Total *n* = 22	UC *n* = 11	Exp *n* = 11
*Age* (years), median (IQR)	63.0 (53.8 to 67)	65.0 (59 to 70)	57.0 (52 to 64)
*Gender*, n male (%)	15 (68)	7 (64)	8 (73)
*Ethnicity, n (%)*			
*White Australian*	18 (82)	9 (82)	9 (82)
*Asian*	2 (9)	1 (9)	1 (9)
*Aboriginal or Torres Strait Islander*	0 (0)	0 (0)	0 (0)
*Other*	2 (9)	1 (9)	1 (9)
*Current work, y (%)*	8 (36)	5 (45)	3 (27)
*Home alone, y (%)*	6 (27)	3 (27)	3 (27)
*BMI* (kg/m^2^), median (IQR)	30.6 (25.8 to 32.5)	28.8 (25.7 to 31.6)	31.7 (25.9 to 34.6)
*Comorbidities*, y (%)			
Musculoskeletal	12 (43)	4 (36)	8 (73)
Cardiovascular	10 (36)	6 (55)	4 (36)
Respiratory	5 (18)	3 (27)	2 (18)
Endocrine	4 (14)	0 (0)	4 (36)
Other	8 (29)	6 (55)	2 (18)
*Time since cancer diagnosis* (months), median (IQR)	6 (3.8 to 12.8)	5.0 (3.0 to 7.0)	9.0 (5.0 to 18.0)
*Time from assessment to transplant (weeks), median (IQR)*	11.9 (9.0 to 16.04)	14.0 (9.0 to 16.43)	12.0 (8.1 to 15.9)
*Cancer Type*			
Multiple Myeloma	20 (91)	11 (100)	9 (82)
Hodgkins Lymphoma	1 (5)	0 (0)	1 (9)
Non-Hodgkins Lymphoma	1 (5)	0 (0)	1 (9)
*Bony lesions*, y (%)	11 (50)	7 (64)	4 (36)
*Current treatment type*			
VRD	14 (50)	7 (64)	7 (64)
VCD	4 (14)	2 (18)	2 (18)
R-ICE	1 (4)	0 (0)	1 (9)
Pembrolizumab	1 (4)	0 (0)	1 (9)
Pomalidomide	1 (4)	1 (9)	0 (0)
Daratuzumab	1 (4)	1 (9)	0 (0)
*ECOG score*, n (%)			
0 = Fully active	5 (23)	3 (27)	2 (18)
1 = Restricted strenuous activity	17 (77)	8 (73)	9 (82)
*GLIM Rating*			
No malnutrition, y (%)	21 (75)	10 (91)	11 (100)
Severe malnutrition, y (%)	1 (4)	1 (9)	0 (0)
*Meeting or exceeding ESPEN estimated daily intake requirements,* y (%)	11 (50)	7 (64)	4 (36)
*Meeting PA guidelines* (COSA), y (%)	6 (27)	2 (18)	4 (36)

*BMI* Body Mass Index, *ECOG* Eastern Cooperative Oncology Group, *GLIM* Global Leadership Initiative on Malnutrition, *ESPEN* The European Society for Clinical Nutrition and Metabolism, *COSA* Clinical Oncology Society of Australia

### Adherence with study protocol

Time available for prehabilitation varied from 2 to 8 weeks. A total of 99 exercise sessions (Mean 9 SD 4 per participant) and 32 nutrition sessions (Mean 4 SD 1 per participant) were completed. Eight participants (72%) attended ≥ 50% of scheduled exercise sessions. The most common reasons for missing sessions were other appointments (16 missed sessions) or being unwell due to treatment-related side-effects (13 missed sessions). Eight participants (72%) attended all nutrition sessions and three participants missed one session each (*n* = 1 work, *n* = 1 unwell, *n* = 1 hospital admission) (Fig. 1). Experimental participants completed 77 (SD 73) minutes of weekly moderate-intensity exercise during prehabilitation.

### Adverse events

No major adverse events resulted from prehabilitation. Two participants declined to wear the physical activity monitor at follow-up due to skin discomfort/irritation at T0. Three participants reported minor adverse events that resolved with rest (delayed muscle soreness *n* = 2, dizziness *n* = 1). Participants experienced, on average, 3 complications post-autoSCT. The most common complications were diarrhea (*n* = 14) and febrile neutropenia (*n* = 11). Five participants experienced disease progression or relapse (Supplementary file 2).

### Effect on primary outcome

A between-group difference of + 141 m (95% CI 24 to 257 m) favoring the experimental group was observed for the primary outcome (T2 6-min walk distance) (Table 2). A between-group difference of + 61 m favoring the experimental group was also observed at T1. The size of the effect (d = 1.4, 95% CI 0.12 to 2.5) exceeded clinical significance [42, 43].

**Table 2:** Mean (SD) of groups, mean (95% CI) difference within groups, and mean (95% CI) difference between groups

Outcome	Groups						Difference within groups				Difference between groups (95%CI)		Standardised mean difference (95%CI)
	Baseline (T0)		Pre-transplant (T1)		1-month post-transplant (T2)		T1 minus T0		T2 minus T0		T1 minus T0	T2 minus T0	T2
	Exp (*n* = 11)	Con (*n* = 11)	Exp (*n* = 11)	Con (*n* = 11)	Exp (*n* = 11)	Con (*n* = 11)	Exp	Con	Exp	Con	Exp-Con	Exp-Con	Exp-Con
6-min walk test (m)	442.3 (383.8 to 500.9)	486.2 (432.6 to 539.8)	518.2 (455.5 to 581.0)	501.5 (443.2 to 559.8)	565.5 (477.4 to 653.6)	468.6 (410.6 to 526.6)	**67.7 (7.6 to 127.8)**	9.6 (−54.3 to 73.0)	136.7 (−110.7 to 384.0)	−17.1 (−127.9 to 93.8)	**60.6 (−40.2 to 161.5)**	**140.8 (24.3 to 257.3)**	**1.4 (0.12 to 2.5)**
Self-Efficacy (0–3)	2.2 (1.8 to 2.6)	2.3 (1.9 to 2.8)	1.9 (1.5 to 2.3)	2.2 (1.8 to 2.6)	1.6 (1.1 to 2.1)	1.9 (1.5 to 2.3)	−0.29 (−0.81 to 0.24)	−0.16 (−0.8 to 0.5)	−0.66 (−1.6 to 0.27)	−0.5 (−0.8 to 0.5)	−0.12 (−0.87 to 0.62)	−0.14 (−0.92 to 0.65)	−0.43 (−1.3 to 0.51)
Weight (kg)	89.0 (87.4 to 90.7)	89.6 (87.9 to 91.2)	90.2 (88.5 to 91.8)	89.2 (87.5 to 90.9)	87.1 (85.0 to 89.3)	85.6 (83.9 to 87.3)	1.1 (−0.63 to 2.9)	−0.4 (−2.2 to 1.4)	−1.8 (−5.9 to 2.4)	−4.0 (−7.2 to −0.8)	1.5 (—1.3 to 4.3)	2.1 (- 1.0 to 5.2)	0.58 (−0.48 to 1.58)
PG-SGA score (0-)	3.0 (0.86 to 5.1)	2.6 (0.56 to 4.7)	3.0 (0.86 to 5.1)	2.1 (0.01 to 4.2)	8.4 (5.8 to 11.0)	8.1 (5.9 to 10.3)	0.00 (−1.2 to 1.2)	−0.55 (−2.3 to 1.2)	5.8 (−0.54 to 12.3)	**5.4 (1.4 to 9.3)**	0.55 (−3.5 to 4.6)	−0.01 (−4.4 to 4.4)	0.09 (−0.88 to 1.05)
Handgrip strength (kg)	39.3 (36.9 to 41.8)	41.2 (38.9 to 43.5)	40.7 (37.8 to 43.7)	41.9 (39.4 to 44.4)	35.5 (31.7 to 39.2)	36.2 (33.7 to 38.7)	2.2 (−1.0 to 5.4)	0.9 (−2.5 to 4.2)	−3.5 (−16.6 to 9.6)	−4.9 (−9.5 to −0.3)	0.73 (−3.9 to 5.4)	1.2 (−4.0 to 6.4)	−0.19 (−1.3 to 0.92)
C-Reactive Protein (mg/L)	8.3 (−4.8 to 21.4)	1.9 (−13.8 to 16.9)	13.9 (0.8 to 27.0)	1.8 (−13.8 17.5)	17.1 (1.6 to 32.4)	1.9 (−16.4 to 20.2)	5.6 (−16.1 to 27.4)	−1.1 (−3.6 to 1.4)	7.7 (−29.6 to 45.0)	−2.0 (−4.2 to 0.2)	5.7 (−21.6 to 33.0)	8.7 (- 21.5 to 38.9)	0.68 (−0.44 to 1.7)
MVPA (minutes)	15.4 (9.7 to 21.1)	14.8 (9.1 to 20.5)	9.5 (3.1 to 15.9)	18.9 (12.9 to 24.8)	5.4 (−1.3 to 12.0)	11.1 (5.2 to (17.0)	**−6.4 (−12.0 to −0.3)**	4.3 (−6.5 to 15.1)	−11 (−23.8 to 1.9)	−3.4 (−10.5 to 3.5)	−10.0 (−1.1 to 21)	- 6.3 (−17.6 to 5.0)	−0.60 (−1.50 to 0.36)
Sedentary time (hours)	19.0 (18.2 to 19.8)	18.6 (17.9 to 19.4)	19.3 (18.4 to 20.1)	18.5 (17.7 to 19.3)	20.6 (19.7 to 21.5)	20.1 (19.3 to 20.9)	0.27 (−0.36 to 0.91)	−0.1 (−1.3 to 1.0)	**1.7 (0.3 to 3.1)**	**1.5 (0.0 to 3.0)**	0.430 (−1.0 to 1.9)	0.2 (−1.6 to 1.3)	0.38 (−0.55 to 1.29)
*EORTC QLQ C30 (0–100)*													
Global	78.7 (70.0 to 87.4)	79.4 (70.7 to 88.1)	77.2 (68.4 to 85.9)	80.2 (71.5 to 88.9)	63.4 (53.2 to 73.6)	60.4 (51.7 to 69.1)	−1.5 (−7.6 to 4.5)	0.8 (−11.9 to 13.4)	**−16.7 (−27.8 to −5.5)**	**−18.9 (−37.2 to −0.7)**	−2.3 (−19.1 to 14.5)	3.7 (−14.0 to 21.3)	0.21 (−0.72 to 1.11)
Physical Function	85.1 (77.1 to 93.1)	88.3 (80.4 to 96.2)	89.3 (81.3 to 97.3)	84.2 (76.2 92.0)	81.7 (72.5 to 90.9)	67.7 (59.8 to 75.6)	4.2 (−6.0 to 14.5)	−4.2 (−8.8 to 0.4)	−4.2 (−23.2 to 14.9)	-**20.6 (−32.0 to −9.2)**	8.5 (−5.7 to 22.7)	**17.2 (2.3 to 32.1)**	**1.07 (0.06 to 1.99)**
Role Function	82.5 (70.5 to 94.5)	86.9 (75.0 to 98.8)	85.6 (73.6 to 97.6)	83.8 (71.9 to 95.8)	76.9 (63.0 to 90.9)	56.6 (44.7 to 68.5)	3.0 (−11.8 to 17.9)	**−3.0 (−12.8 to −8.8)**	−4.2 (−32.8 to 24.4)	**−30.3 (−52.0 to −8.6)**	6.1 (−16.5 to 28.6)	**24.7 (1.1 to 48.3)**	**1.03 (0.02 to 1.95)**
Emotional Function	87.1 (78.2 to 95.9)	86.5 (77.8 to 95.2)	85.5 (76.7 to 94.4	88.9 (80.3 to 97.6)	88.3 (78.2 to 98.4)	85.0 (76.3 to 93.7)	−1.5 (−9.4 to −11.8)	3.0 (−7.8 to 12.3)	2.1 (−8.3 to 12.5)	−1.5 (−20.2 to 17.2)	−3.924 (−19.3 to 11.4)	2.8 (−13.4 to 18.9)	0.23 (−0.69 to 1.14)
Cognitive function	83.7 (73.7 to 93.7)	85.6 (75.7 to 95.6)	83.698 (73.7 to 93.7)	88.7 (78.7 to 98.6)	87.3 (75.7 to 99.0)	76.6 (66.6 to 86.5)	0 (−10.0 to 10.0)	3.0 (−8.0 to 14.0)	4.2 (−2.3 to 10.6)	−9.1 (−30.5 to 12.4)	−3.0 (−22.5 to 16.4)	12.7 (−7.6 to 33.0)	0.65 (−0.31 to 1.6)
Social Function	79.2 (68.5 to 90.0)	83.0 (72.3 to 93.7)	80. 8 (70.0 to 91.5)	84.5 (73.8 to 95.226	71.0 (58.5 to 83.6)	61.8 (51.0 to 72.5)	1.5 (−17.5 to 20.6)	1.5 (−7.8 to 10.8)	−8.3 (−34.1 to 17.5)	**−21.2 (−34.5 to −7.9)**	(0.00 −20.9 to 20.9)	13.0 (−8.8 to 34.8)	0.52 (−0.43 to 1.4)
Fatigue	29.3 (19.5 to 39.3)	27.2 (17.4 to 37.1)	32.4 (22.5 to 42.3)	22.2 (12.3 to 32.0)	46.0 (34.4 to 57.5)	46.4 (36.6 to 56.3)	3.0 (−5.9 to 11.9)	−5.1 (−12 to 1.9)	16.7 (−11.4 to 44.8)	**19.2 (5.8 to 32.6)**	8.1 (−11.1 to 27.2)	−2.6 (−22.6 to 17.5)	−0.03 (−0.94 to 0.88)
Pain	20.8 (11.2 to 30.4)	20.3 (10.7 to 29.9)	23.8 (14.2 to 33.4)	15.8 (6.2 to 25. 4)	11.0 (−0.21 to 22.3)	12.8 (3. 2 to 22. 4)	3.03 (−17.5 to −5.9)	−4.5 (−13.3 to 4.2)	−10.4 (−28.6 to 7.7)	−7.6 (−19.2 to 4.0)	7.6 (−11.1 to 26.2)	−2.2 (−21.7 to 17.3)	−0.11 (−1.0 to 0.81)
Dyspnoea	19.0 (7.8 to 30.2)	20.3 (9.1 to 31.5)	6.8 (−4.4 to 18.0)	17.3 (6.1 to 28.4)	10.0 (−3.1 to 23.0)	23.3 (12.2 to 34.5)	**−12.1 (−23.4 to −0.8)**	−3.0 (−21.6 to 15.6)	−8.3 (−28.0 to 11.4)	3.0 (−18.1 to 24.2)	−9.1 (−30.8 to 12.6)	−12.0 (−34.7 to 10.7)	−0.72 (−1.6 to 0.25)
Insomnia	23.1 (7.4 to 38.8)	27.7 (12.0 to 43.3)	26.2 (10.5 to 41.8)	24.7 (9.0 to 40.3)	39.1 (20.4 to 57.6)	21.6 (5.9 to 37.3)	3.0 (−15.6 to 21.6)	−3.0 (−24.2 to 18.1)	20.8 (−21.1 to 62.8)	−6.1 (−34.1 to 21.9)	6.1 (−24.2 to 36.3)	22.0 (−9.8 to 53.7)	0.67 (−0.29 to 1.6)
Appetite	7.3 (−6.5 to 21.1)	4.7 (−9.1 to 18.4)	10.3 (−3.5 to 24.1)	4.7 (−9.1 to 18.4)	27.4 (11.3 to 43.5)	31.9 (18.2 to 45.7)	3.0 (−9.0 to 15)	1.5 (−4.0 to 7.1)	20.8 (−4.7 to 46.4)	27.3 (−2.5 to 57.0)	−3.0 (−23.7 to 29.77)	−7.2 (−35.1 to 20.8)	−0.20 (−1.1 to 0.72)
*BMT-29 (0–100)*													
Gastrointestinal symptoms	12.6 (7.8 to 17. 3)	12.1 (7.4 to 16.8)	9.5 (4.8 to 14.3)	10.3 ( 5.5 to 14.9)	15.7 (10.2 to 21.2)	18.7 (14.0 to 23.4)	−3.0 (−8.1 to 2.0)	−1.8 (−6.3 to 2.7)	3.3 (−10.0 to 16.7)	**6.7 (0.0 to 13.3)**	−1.2 (−10.2 to 7.9)	−3.5 (−13.0 to 5.9)	−0.39 (−1.3 to 0.54)
Anxiety	16.8 (8.2 to 25.3)	17.0 (8.5 to 25.4)	17.8 (9.3 to 26.3)	18.8 (10.4 to 27.2)	21.9 (12.2 to 31.7)	22.3 (13.8 to 30.7)	1.0 (−5.2 to 7.2)	2.0 (−5.3 to 9.3)	5.6 (−16.5 to 27.6)	5.3 (−4.6 to 15.2)	−0.84 (−12.8 to 14.5)	−0.15 (−14.5 to 14.2)	−0.02 (−0.93 to 0.89)
Family	30.2 (21.0 to 39.4)	25.73 (16.6 to 34.9)	24.9 (15.7 to 34.1)	28.8 (19.7 to 37.9)	32.4 (21.7 to 43.1)	40.3 (31.0 to 49.3)	−5.3 (−21.6 to 11.)	3.0 (−15.6 to 21.6)	4.2 (−15.2 to 23.5)	**14.4 (3.5 to 25.3)**	8.4 (−26.1 to 9.4)	−12.2 (−30.8 to 6.4)	−0.51 (−1.4 to 0.44)
Body Image	15.7 (4.6 to 26.9)	13.4 (2.3 to 24.4)	20.3 (9.1 to 31.5)	8.8 (−2.2 to 19.9)	32.3 (19.4 to 45.3	28.5 (17.4 to 39.6)	4.5 (−4.3 to 13.3)	−4.5 (−11.8 to 2.7)	16.7 (−4.4 to 37.7)	15.2 (−5.8 to 36)	9.1 (−11.5 to 29.7)	1.4 (−20.1 to 23.0)	0.21 (−0.71 to 0.44)
Ache	20.0 (8.0 to 31.9)	21.7 (9.9 to 33.6)	35.1 (23.1 to 47.0)	24.8 (12.9 to 36.7)	10.0 (−3.9 to 24.0)	15.7 (3.8 to 27.6)	15.2 (−3.2 to 33.5)	3.0 (−15.6 to 21.6)	−8.3 (−33.0 to 16.4)	−6.1 (−25.6 to 13.5)	12.1 (−10.8 to 35.0)	−3.8 (−27.8 to 20.1)	−0.29 (−1.2 to 0.64)
Life	59.2 (39.9 to 78.5)	38.3 (19.2 to 57.4)	62.2 (42.9 to 81.5)	56.5 (37.4 to 75.5)	62.6 (40.5 to 84.8)	47.4 (28.3 to 66.4)	3.0 (−22.4 to 28.5)	18.2 (−10.8 to 47.1)	8.3 (−40.5 to 57.2)	9.1 (−13.5 to 31.7)	−15. 2 (−51.9 to 21.6)	−5.6 (−44.2 to 33.0)	0.49 (−0.46 to 0.48)

*NB* Means adjusted for baseline differences in age, time since diagnosis and outcome for between group analyses; Bold indicates significance at *p* < 0.05

### Effect on secondary patient outcomes

Large between-group differences favoring the experimental group were observed for physical (17 points, 95% CI 2 to 32) and role function (25 points, 95% CI 1 to 48) subscales on the EORTC-QLQ C30 at T2. No other secondary outcome achieved significance. However, moderate-to-large effects favoring the experimental group at T2 were observed for higher body weight (+ 2 kg, 95% CI −1 to 5.2), improved cognitive (+ 13 points, 95% CI −8 to 33), social function (+ 13 points, 95% CI −9 to 35), being able to distinguish what is important in life (−6 points, 95% CI −44 to 33 points) and reduced dyspnea (−12 points, 95% CI −35 to 11) and gastrointestinal symptoms (−3.5 points, 95% CI −13 to 5.9).

Participants in the experimental group were not more likely to achieve physical activity [39] (RR 0.972, 95% CI 0.694 to 1.357) or nutritional intake recommendations [24] (RR 0.917, 95% CI 0.381 to 2.206) than the usual care group (supplementary file 3). Calcium was the most deficient micronutrient (*n* = 3, 17%).

### Effect on secondary health service outcomes

Time to stem cell engraftment was similar between groups (z = −0.423, *p* = 0.672) (Table 3).

**Table 3:** Health service outcomes

	Experimental Group *n* = 9	Control Group *n* = 11
Hospital Length of Stay *Median (IQR), days*	15 (14 to 23)	17 (13 to 19)
Time to engraftment *Median (IQR), days*	19 (16 to 20)	18 (16 to 19)
ED presentations *number of participants (%)*	1 (11)	2 (18)
Hospital readmissions *number of participants (%)*	1 (11)	1 (9)
SURC presentations *number of participants (%)*	3 (33)	3 (27)

*NB* *N* = denotes number of participants. Only one participant in the experimental group contacted the SURC clinic 4 times

There was no between-group difference in hospital length of stay (z = −0.343, *p* = 0.731). Two participants in the control group and one in the experimental group presented to the emergency department and one patient in each group was readmitted to hospital in the 3 months following transplant (Table 3).

## Discussion

This randomized controlled trial found multidisciplinary prehabilitation for people awaiting autoSCT was safe, feasible and may result in better walking capacity. Large estimates of effect were observed for self-reported function, higher body weight and less dyspnea and gastrointenstinal symptoms at 1-month post-transplant compared to usual care. There was uncertainty regarding whether prehabilitation had a positive effect on overall quality-of-life or health service outcomes. This is the first pilot trial to evaluate combined exercise and nutrition prehabilitation for autoSCT, with results justifying a larger full-scale trial to confirm effectiveness.

This trial establishes safety of multidisciplinary prehabilitation, aligning with previous evidence supporting exercise prehabilitation [9, 44]. This is important given this population may be more vulnerable than other cancer cohorts when participating in physical rehabilitation due to high dose chemotherapy and disease processes causing immunocompromise, bony disease with potential instability and hemodynamic instability [45, 46]. The safety of prehabilitation may be attributed to guideline-informed design of assessment and intervention delivered by trained clinicians [6, 18, 47]. These guidelines help provide a framework for decision making about participant suitability and tailoring prehabilitation interventions to optimize safety.

The feasibility of multidisciplinary prehabilitation was indicated by high recruitment, (81% of eligible participants) retention (87%) and attendance to sessions. These rates were much higher than previous studies (e.g. 46%−56% recruitment, 57–60% retention at discharge [48, 49]). In our trial, the clinical nurse coordinator was critical for ensuring patients attending the haematology clinic were aware of the study by following *‘ask, assess, refer’* recommendations in their transplant education sessions [39, 50].

Acceptability of the trial may have been positively influenced by the home-based setting. Typically, rehabilitation interventions (including prehabilitation), are provided in a health facility [51, 52]. However, this patient cohort have many competing medical demands in preparation for their procedure [49, 53]. Home-based therapy provides convenience and lower treatment burden [54, 55] to overcome these issues although the appropriateness of home-based therapy for patients with unstable bone disease needs to be considered. Participants with unstable bony lesions require high levels of supervision by an exercise professional and may be better suited to being treated within a health facility where the specialist treating team available to communicate with exercise professionals and patients about their capacity to exercise safely [47]. Telehealth can also overcome many participation barriers and is a feasible and effective alternative to face-to-face care [56]. The addition of telehealth for nutrition and some exercise sessions because of COVID-19 restrictions allowed participants to continue with rehabilitation during a vulnerable time.

Preliminary evidence of efficacy of multidisciplinary prehabilitation was demonstrated by positive estimates of effect for improved walking capacity increased body weight and self-reported function in experimental participants. This finding supports results from a recent review about exercise before and after SCT [9], and recent feasibility studies of exercise prehabilitation for people with multiple myeloma [48, 49]. AutoSCT results in long term impairments to physical function over 1 year after transplant, suggesting natural recovery of premorbid function doesn’t naturally occur [57]. Maintenance of physical function after autoSCT is also important given its relationship with premature death and unplanned hospital admission in people with hematological malignancies [58].

The nutrition intervention may explain other benefits of prehabilitation not detected in previous studies, such as weight gain and lower gastro-intestinal symptoms. Optimising nutritional outcomes is essential in transplant settings given patients are at high risk of malnutrition before, during and after transplant [59]. Malnutrition and transplant-related weight loss is highly correlated with longer hospital admissions, time to platelet engraftment, infection and 1-year mortality [59]. Therefore, even small changes in weight gain may be clinically significant. The combination of exercise with nutrition may have compounding effects to build functional reserve and explain positive effects found in this study, contrasting with a similar recent trial [53] that did not have a dietary component.

Similar to another recent trial [48], no differences in health service outcomes were observed despite benefits in patient outcomes. This finding contradicts previous literature that prehabilitation may reduce hospital length of stay [9]. It is possible that the current trial was underpowered to detect an effect in hospital outcomes given the small sample size. However, considering the complexity of transplant recovery and many factors contributing to hospital length-of-stay including medical complications, prehabilitation alone may be unable to overcome these issues. There was a very low rate of hospital readmission in both groups, with reasons for readmission being related to medical treatment. Therefore, once patients are medically stable after initial acute recovery, the risk of modifiable reasons for hospital readmission in the short-term (such as falls) appears to be very low.

### Strengths and limitations

This is the first study to demonstrate positive effects of multidisciplinary prehabilitation for people preparing for autoSCT. This study, which had high recruitment rate, was conducted in a pragmatic public hospital setting and included patients who have previously been excluded from prehabilitation research such as those with refractory disease and bony lesions, increasing generalizability of findings.

There were several limitations. Ongoing COVID-19 restrictions throughout the trial resulted in a relatively high amount of missing data, particularly for our primary outcome. However, the impact of these extenuating circumstances was reported in alignment with recommendations [60] and linear mixed effects-modelling was used to account for missing data. There was wide variability in our outcomes as a result of the small sample size. There may also be an overestimate of the effect of our primary outcome due to Type I error and baseline imbalance in age and time since diagnosis. However, results align with previous research [48, 49] and positively correlate with self-reported function of which there was full data available and baseline parity in most outcomes. It should also be acknowledged that tri-modal cancer prehabilitation, including psychology interventions such as stress management, is commonly recommended [61]. Given the small, pragmatic nature of the trial, additional psychological interventions were unable to be included but warrant further investigation in a future larger trial.

This trial provides preliminary evidence multidisciplinary prehabilitation may improve function and is safe and feasible after autoSCT. Findings support a larger trial to confirm the effectiveness of multidisciplinary prehabilitation. Given the small numbers of autoSCT recipients at most individual tertiary centers, a full-scale trial would need to be multi-site to obtain a sufficient sample size. Successful recruitment in this trial was heavily dependent on a pro-active clinical nurse coordinator and home-based setting suggesting these strategies should be adopted for a future trial.

## Supplementary Information

Below is the link to the electronic supplementary material.

Supplementary file1 (DOCX 36 KB)

## Data Availability

The dataset generated during and/or analysed during the current study are available from the corresponding author on reasonable request.
