# Supplementary material for: Prehabilitation to improve function after autologous stem cell transplantation: A pilot randomized controlled trial (PIRATE)
Source: Support Care Cancer. 2025 Feb 7;33(3):164. doi: 10.1007/s00520-025-09179-1 (PMC11805779; doi:10.1007/s00520-025-09179-1)
Supplement: Supplementary file 1 — Supplementary file1 (DOCX 36 KB) [file 520_2025_9179_MOESM1_ESM.docx]

|  | **Experimental Group** | | **Control Group** |
| --- | --- | --- | --- |
| **Brief Name** | Prehabilitation | | Usual Care |
| **Why** | Prehabilitation may build functional reserves to better cope with transplant | | Pragmatic trial design |
| **What: Materials** | - Participants will use combination of hospital-owned and their own exercise equipment: - Free weights - Resistance exercise bands - Portable stepper - Participants will receive 1) standardised written handout exercise 2) standardised written handout nutrition 3) referral to Oncology Rehabilitation Program post-transplant - Participants will receive a Fitbit Inspire device worn continuously at the wrist for the duration of their prehabilitation period - Participants will receive usual hospital care | | - Participants will receive 1) standardised written handout exercise 2) referral to Oncology Rehabilitation Program post-transplant - Usual care also includes usual medical care which may include adjuvant chemotherapy, radiotherapy, inpatient admission post-transplant, specialist, nursing and allied health outpatient appointments, visits to their general practitioner and general advice from their medical team to remain active and eat a healthy diet. |
| **What Procedures** |  | | |
| **Provider** | Physiotherapist and dietitian with oncology experience provided by the hospital | | Usual hospital staff |
| **How** | Face to face sessions +/- telehealth (pending COVID-19 lockdown directives) | | No intervention |
| **Where** | Patient’s home | | No intervention |
| **When/How much**  **Intensity**  **Frequency**  **Session time**  **Overall duration** | **Exercise** | ***Nutrition*** | - Standardised written advice about exercise and cancer guidelines |
|  | Moderate (BORG 4-6)  60-80% heart rate maximum  10-12 repetition maximum |  |  |
|  | 2x weekly supervised  1X weekly unsupervised | Fortnightly |  |
|  | 60 minutes | 30 minutes |  |
|  | Up to 8 weeks | |  |
| **Tailoring** | - Individualised exercise program and nutrition advice based on initial consultation and goals | | - None |
| **Trial fidelity** | - Staff with a background in oncology physiotherapy and dietetics who had prior formal training were employed by the hospital to provide the intervention - Exercise log-books will be completed and reviewed by research staff. - Nutrition log-books will be completed and reviewed by research staff. - Records of the number and duration of completed sessions. - Monthly meetings with clinical research staff | | - Participants will be asked if they participated in any physical activity or nutrition intervention during the usual care period. |

Supplementary File 1. Intervention description using the template for description and replication checklist (TIDier)

Supplementary File 2. Frequency of transplant complications

|  | *Control group n=11* | *Experimental Group (n=9)* |
| --- | --- | --- |
| Diarrhea | 10 (91%) | 4 (44%) |
| Febrile neutropenia | 7 (64%) | 4 (44%) |
| Mucositis | 2 (18%) | 2 (22%) |
| Rash | 1 (9%) | 0 |
| Fever | 1 (9%) | 1 (11%)+ |
| Nausea | 0 | 1 (11%) |
| Neutropaenic sepsis | 1 (9%) |  |
| Hypotension | 1 (9%) |  |
| Electrolyte derangement | 1 (9%) | 2 (22%) |
| LFT Derangement | 1 (9%) |  |
| Oral Thrush | 1 (9%) | 1 (11%) |
| DVT | 0 | 1 (11%)+ |
| Engraftment syndrome | 0 | 1 (11%)+ |
| Slow atrial fibrillation | 0 | 1 (11%) |
| Tachycardia | 0 | 1 (11%) |
| Acute kidney injury | 0 | 1 (11%)+ |
| Vancomyocin reaction | 0 | 1 (11%) |
| Delerium | 0 | 1 (11%)+ |
| Hypertriglyceridemia | 0 | 1 (11%)+ |
| Severe COVID Pneumonitis | 0 | 1 (11%)+ |
| Gastrointestinal bleed | 0 | 1 (11%)+ |
| Aspiration pneumonia | 0 | 1 (11%)+ |

*Patients may have experienced one or more complications

+ complications experienced by a single patient admitted to ICU

Supplementary File 3. Dietary outcomes from the Australian Eating Survey

|  | Baseline (T0) | | 1-month post-transplant (T2) | |
| --- | --- | --- | --- | --- |
|  | Exp n=11 | Con n=10 | Exp n=8 | Con n=11 |
| Estimated Daily energy intake *KJ/day* | 11689  (4918 to 18461) | 9943  (7557 to 12328) | 9584  (3834 to 15334) | 10966  (6850 to 15081) |
| Carbohydrate  *% of estimated total energy expenditure* | 44 (37 to 51) | 42 (36 to 48) | 46 (41 to 51) | 47 (42 to 51) |
| Protein  *% of estimated total energy expenditure* | 19 (15 to 24) | 19 (16 to 21) | 19 (16 to 22) | 18 (16 to 20) |
| Fat  *% of estimated total energy expenditure* | 37 (32 to 41) | 38 (34 to 42) | 35 (33 to 37) | 34 (31 to 37) |
| *Comparison with ESPEN Guidelines^23^* | |  |  |  |
| Meeting estimated intake requirements^23^  *Yes %* | 4 (36) | 7 (70) | 4 (50) | 6 (55) |
| Meeting estimated protein requirements^23^  *Yes %* | 10 (91) | 9 (90) | 7 (88) | 11 (100) |

Control group missing baseline data n=1
